# Utilizing native fluorescence imaging, modeling and simulation to examine pharmacokinetics and therapeutic regimen of a novel anticancer prodrug

**DOI:** 10.1186/s12885-016-2508-6

**Published:** 2016-07-25

**Authors:** Jing-Hung Wang, Aaron N. Endsley, Carol E. Green, A. C. Matin

**Affiliations:** 1 Department of Microbiology and Immunology, Stanford University School of Medicine, Sherman Fairchild Science Building, 299 Campus Drive, Stanford, CA 94305 USA; 2 Bioanalytical Assays and Pharmacokinetics, Bayer HealthCare LLC, 455 Mission Bay Boulevard South, San Francisco, CA 94158 USA; 3 Biosciences Division, SRI International, Menlo Park, 94025 CA USA

**Keywords:** CNOB, Prodrug, Cancer, Fluorescence, Imaging, Pharmacokinetics, Modeling and simulation

## Abstract

**Background:**

Success of cancer prodrugs relying on a foreign gene requires specific delivery of the gene to the cancer, and improvements such as higher level gene transfer and expression. Attaining these objectives will be facilitated in preclinical studies using our newly discovered CNOB-GDEPT, consisting of the produrg: 6-chloro-9-nitro-5-oxo-5H-benzo-(a)-phenoxazine (CNOB) and its activating enzyme ChrR6, which generates the cytotoxic product 9-amino-6-chloro-5H-benzo[a]phenoxazine-5-one (MCHB). MCHB is fluorescent and can be noninvasively imaged in mice, and here we investigated whether MCHB fluorescence quantitatively reflects its concentration, as this would enhance its reporter value in further development of the CNOB-GDEPT therapeutic regimen. PK parameters were estimated and used to predict more effective CNOB administration schedules.

**Methods:**

CNOB (3.3 mg/kg) was injected iv in mice implanted with humanized ChrR6 (HChrR6)-expressing 4T1 tumors. Fluorescence was imaged in live mice using IVIS Spectrum, and quantified by Living Image 3.2 software. MCHB and CNOB were quantified also by LC/MS/MS analysis. We used non-compartmental model to estimate PK parameters. Phoenix WinNonlin software was used for simulations to predict a more effective CNOB dosage regimen.

**Results:**

CNOB administration significantly prolonged mice survival. MCHB fluorescence quantitatively reflected its exposure levels to the tumor and the plasma, as verified by LC/MS/MS analysis at various time points, including at a low concentration of 2 ng/g tumor. The LC/MS/MS data were used to estimate peak plasma concentrations, exposure (AUC_0-24_), volume of distribution, clearance and half-life in plasma and the tumor. Simulations suggested that the CNOB-GDEPT can be a successful therapy without large increases in the prodrug dosage.

**Conclusion:**

MCHB fluorescence quantifies this drug, and CNOB can be effective at relatively low doses. MCHB fluorescence characteristics will expedite further development of CNOB-GDEPT by, for example, facilitating specific gene delivery to the tumor, its prolonged expression, as well as other attributes necessary for successful gene-delivered enzyme prodrug therapy.

**Electronic supplementary material:**

The online version of this article (doi:10.1186/s12885-016-2508-6) contains supplementary material, which is available to authorized users.

## Background

Cancer prodrugs are typically small molecules that are essentially nontoxic but can be converted to a cytotoxic compound (referred to from hereon as the “drug”) by enzyme-catalyzed reactions [1]. A class of these (the “N-prodrugs”) relies on enzymes native to humans, which are expressed at a higher level in malignant compared to normal cells. An example of this class is Mitomycin C (MMC), which is reductively activated by nitroreductases, particularly the mammalian NQO1, whose concentration is up-regulated in cancer cells [2, 3], making them more vulnerable to its action. However, as the normal cells also produce such enzymes, they too activate MMC, resulting in serious off-target toxicity with this and other N-prodrugs.

Another class of prodrugs (the “F-prodrugs”) requires targeting to tumors of a foreign gene that encodes the enzyme needed to generate the drug. This approach is referred to as gene-delivered enzyme prodrug therapy (GDEPT). It holds the promise of largely avoiding off-target toxicity if the delivery of the gene and activation of the prodrug are confined specifically to the tumor, and considerable effort has been underway to develop this therapeutic approach [4–7]. The prodrug Genciclovir (GC), which is activated by the herpes simplex virus 1 thymidine kinase (TK1), was examined in a 4-year Phase III clinical trial involving 248 glioblastoma multiforme patients [5]; and another prodrug, 5-aziridinyl-2,4-dinitrobenzamide (CB1954), which requires the *Escherichia coli* nitroreductase enzyme (NTR), is in clinical trial for prostate cancer [8]. These studies have indicated that the success of GDEPT depends, apart from the obvious importance of specificity of gene delivery to cancer, also on: a) high level gene transfer; b) extended duration of gene expression; c) increasing the potency of the activating enzyme; and d) an efficient bystander effect (BE). (BE refers to the spread of the activated drug from the transformed cells capable of producing it to the neighboring cells lacking this capacity, and is critical to the efficacy of any GDEPT therapy because no method of gene delivery can transform all the cancer cells in a tumor.)

Attaining these objectives would be facilitated by a prodrug regimen whose drug product could be visualized non-invasively in living mice, as the resulting ‘observational approach’ would minimize the need for mouse sacrifice and the use of more involved tests, such as LC/MS/MS; native fluorescence in a drug is also superior to attaching a fluorophore to visualize it, as the fluorophore may affect the drug in unpredictable ways [9].

We have previously reported the discovery of such a regimen [10, 11], consisting of the prodrug 6-chloro-9-nitro-5-oxo-5H-benzo-(a)-phenoxazine (CNOB), and the newly discovered bacterial nitroreductase (also referred to as chromate reductase), ChrR. We have improved the latter several-fold, generating ChrR6 and its humanized version HChrR6 [12, 13]. The activated cytotoxic product of CNOB, 9-amino-6-chloro-5H-benzo[a]phenoxazine-5-one (MCHB), is fluorescent and has been successfully visualized non-invasively in living mice; this is illustrated in Additional file 1: Figure S1 (reproduced from reference [11] for convenience). The figure shows that in tumors producing ChrR6, MCHB is visible following iv CNOB injection, but not in tumors lacking ChrR6 [11]. The CNOB/ChrR6 regimen (referred to from hereon as ‘CNOB-GDEPT’) is effective not only in killing several different cancer cell lines in vitro, but also in treating implanted 4T1 murine mammary tumors in mice with 40 % complete survival on day 140 (10 mg/kg CNOB administered in three daily doses of 3.3 mg/kg); all the untreated mice in this study were dead by day 25 [11]. These 4T1 tumors represent human stage IV breast cancer model, reflective of both disease progression and metastatic characteristics [14]. CNOB alone, even at high concentrations (up to 20 mg/kg), showed no significant toxicity, as determined by blood chemistry panel values. MCHB has an impressive BE and kills cells by intercalating with mitochondrial DNA, causing apoptosis involving the mitochondrial pathway, and likely kills both growing and non-growing cells [11].

Our previous work established that CNOB fluorescence indicated its presence, but to what degree the fluorescence represented MCHB quantity was not addressed. As quantitative representation of MCHB by its fluorescence would enhance its utility in preclinical studies, we have investigated this here. We report that MCHB fluorescence does quantitatively correspond to its concentration; we also provide information on aspects of the pharmacokinetics (PK) of the CNOB-GDEPT and predictions on more effective CNOB dosages.

## Methods

### Construction of 4T1/HChrR6 cells

4T1 cells (ATCC) were transfected with humanized *chrR6* (*HchrR6*) gene using *Sleeping Beauty* transposon method as described before [11]. Briefly, *HchrR6* gene was cloned into pKT2/UXbG using *Hin*dIII/*Apa*I restriction sites, creating pKT2/hU-*HchrR6*-SN. Cells were grown to 90–95 % confluence in DMEM without antibiotics in a six-well plate. Transposase vector (pUb-SB11; 0.8 μg) and transposon DNA (pKT2/hU-*HchrR*-SN and pKT2/BGL; 7.2 μg) were added to 0.5 mL Opti-MEM (Invitrogen). In another vial, 20 μL of Lipofectamine 2000 (Invitrogen) were added to 0.5 mL of Opti-MEM, and incubated at room temperature (5 min). The medium was aspirated and cells were washed once with PBS. The above solutions were combined, added to each well (total of 1 mL/well), and incubated for 18 to 24 h. The transfection solution was then aspirated and replaced by complete DMEM. Cells were incubated for an additional 48 h and selected with geneticin (Invitrogen; 2 mg/mL; this concentration was predetermined as the minimal killing dose for 4T1 cells). To ensure homogeneity of *HchrR6* expression, cells expressing luciferase were diluted to ~30 cells per 10 mL DMEM, supplemented with geneticin, and 100 μL aliquots were dispensed into a 96-well plate. This dilution generates a ~30 % probability of a well receiving a single cell, so that colonies in a well would develop from a single cell.

### In vitro cell viability and fluorescence assays

4T1 cells transfected to constitutively express HChrR6 (‘4T1/HChrR6’ cells) were incubated (37 °C) with 15 μM CNOB for the specified time periods. MCHB fluorescence was measured as described below. Viability was determined at corresponding time periods by the MTS assay.

### *In vivo* studies

Female nude (*nu/nu*) mice were inoculated subcutaneously in mammary fat pad number 9 with 4T1/HChrR6 cells, i.e., cells endogenously generating HChrR6 (1 × 10^6^ cells in 50 % PBS/50 % matrigel). Tumors were allowed to grow for 10-14 days before injecting CNOB (3.3 mg/Kg) and subsequent imaging and fluorescence- or LC/MS/MS-based quantification of MCHB. To minimize background fluorescence, mice were fed purified rodent diet (AIG093, Dyets Inc.). Tumor burden was measured by caliper.

For detecting off-target activation of CNOB, firefly luciferase (F-Luc)-expressing untransfected 4T1 cells (not generating HChrR6) were used for tumor implantation and the tumors were visualized and imaged 5 min after intraperitoneal (ip) injection of luciferin (150 μL of 30 mg/mL solution); the (*luc* promoter*-*controled) *chrR6* gene was delivered using SL7838-*chrR6* bacteria [11, 15]; the bacteria contained the Lux operon, permitting their visualization, as before [11]. The bacteria were treated with IPTG to induce the enzyme before tail vain injection.

### Imaging

Ninety-six-well black color plates with transparent bottom (Costar) and a plate reader (SpectraMax, Molecular Devices) were used for in vitro fluorescence imaging. For *in vivo* experiments, mice carrying 4T1 tumors were injected with CNOB (3.3 mg/kg iv) prior to imaging. The images were acquired by IVIS Spectrum (Perkin Elmer Inc.) and quantified by Living Image 3.2 software (Perkin Elmer Inc.). The exposure time for photography was 1 s. A standard curve was constructed in vitro based on the quantified photon intensity at various MCHB concentrations (0, 1.5, 15, 150, 1500 μM). Bioluminescence was measured using the same instrument. We note that the IVIS instrument is widely used for quantitative fluorescence/bioluminance imaging, as it possesses a built-in calibration system. As already stated, this permitted generation of a standard curve that linearly related MCHB photon yield with its concentration. The imaging experiments were performed at least four times.

### LC/MS/MS analysis

Tumor tissue was weighed and homogenized (using 3x volume of PBS/unit weight) using Pro250 homogenizer (Pro Scientific Inc.). For the preparation of standards, various freshly prepared CNOB/MCHB combinations (0, 0.25, 0.5, 1, 2, 2.5, 5, 10, and 20 ng of each) were mixed either with 0.2 mL of blank tumor homogenate, or spiked to the blank plasma. All samples were further spiked with 25 μL of fresh internal standard (Chembridge ID 6066331) plus 10 μL of NH_4_OH (100 mM), and extracted in 2 mL of ethyl acetate (vortexing and centrifugation at 1400 × g for 10 min). Samples in ethyl acetate phase were evaporated and re-constituted in acetonitrile for LC/MS/MS analysis. Compounds were separated and quantified by the Micromass Quattro Premier triple quadrupole HPLC-MS by experts at Stanford University Mass Spectrometry Lab. The extraction efficiency was 95-99 %. Samples were stored at -80 °C and the LC/MS/MS analysis was performed within 2 weeks; CNOB and MCHB remained stable during this time.

### Prediction of alternate modes of administration and statistical analysis

The Phoenix WinNonlin software (version 6.3, Certara, Princeton, NJ) was used to make projections using the PK data obtained here for predicting a potentially more effective dosage regimen of CNOB-GDEPT. All data were calculated and analyzed by the GraphPad Prism software. Statistics were determined using Student’s *t*-test and correlation analysis; *p* values of less than 0.05 were considered significant.

The statistical analysis of the AUC data (Table 1) was done by log-transformed raw AUC data, followed by two-tail paired *t*-test between groups of two tumor types. The log transformation gives more normally distributed data that better fit the assumptions of the *t*-test. This method is recommended by the US FDA for analyzing AUC of bioequivalent drugs. The reason the *t*-test instead of the z-test (as done for most bioequivalent studies) was used is because of the sample size (<30).

## Results

### Noninvasive visualization of off-target CNOB activation

**Fig. 1:**
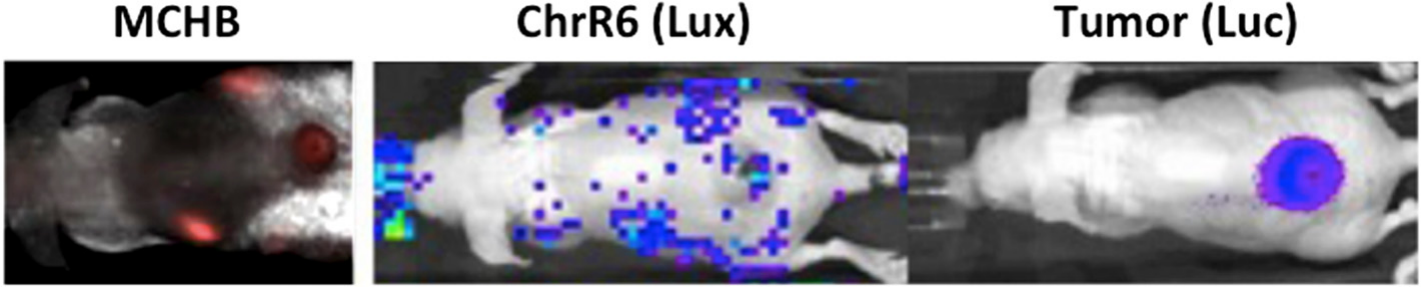
Detection of off-target activation of CNOB by non-invasive imaging. Tumors were implanted in mice using F-Luc expressing non-transfected breast cancer cells that did not generate HChrR6, and the HchrR6 gene was delivered via SL7838-chrR6 bacteria (tail vein injection). Right, center and left pictures show, respectively: the tumor location imaged 5 min after ip injection of luciferin (150 μL of 30 mg/mL); the location of SL7838-chrR6 bacteria in the mice 24 h post iv injection visualized by their Lux expression; and the location of MCHB generation imaged 8 h post tail vein CNOB injection

In our previous work (Additional file 1: Figure S1), activation of CNOB was confined to the implanted breast tumor by injecting ChrR6-generating SL7838 bacteria directly into the tumor. To determine if MCHB fluorescence can permit non-invasive imaging of off-target activation of CNOB, we used similar breast tumor implants and supplied the (*lac* promoter-controlled)-*chrR6* gene for CNOB activation using the above-mentioned ChrR6-generating bacteria injected via the tail vein. We have previously shown that although six days following such injection, bacteria localize exclusively in the tumors, they initially colonize other organs as well [11, 15]. At 24 h, the SL7838 bacteria, visualized by Lux expression, did indeed show a wide distribution but with concentration in kidneys and the tumor; MCHB could be non-invasively visualized at both sites (Fig. 1).

### Quantitative nature of MCHB fluorescence

**Fig. 2:**
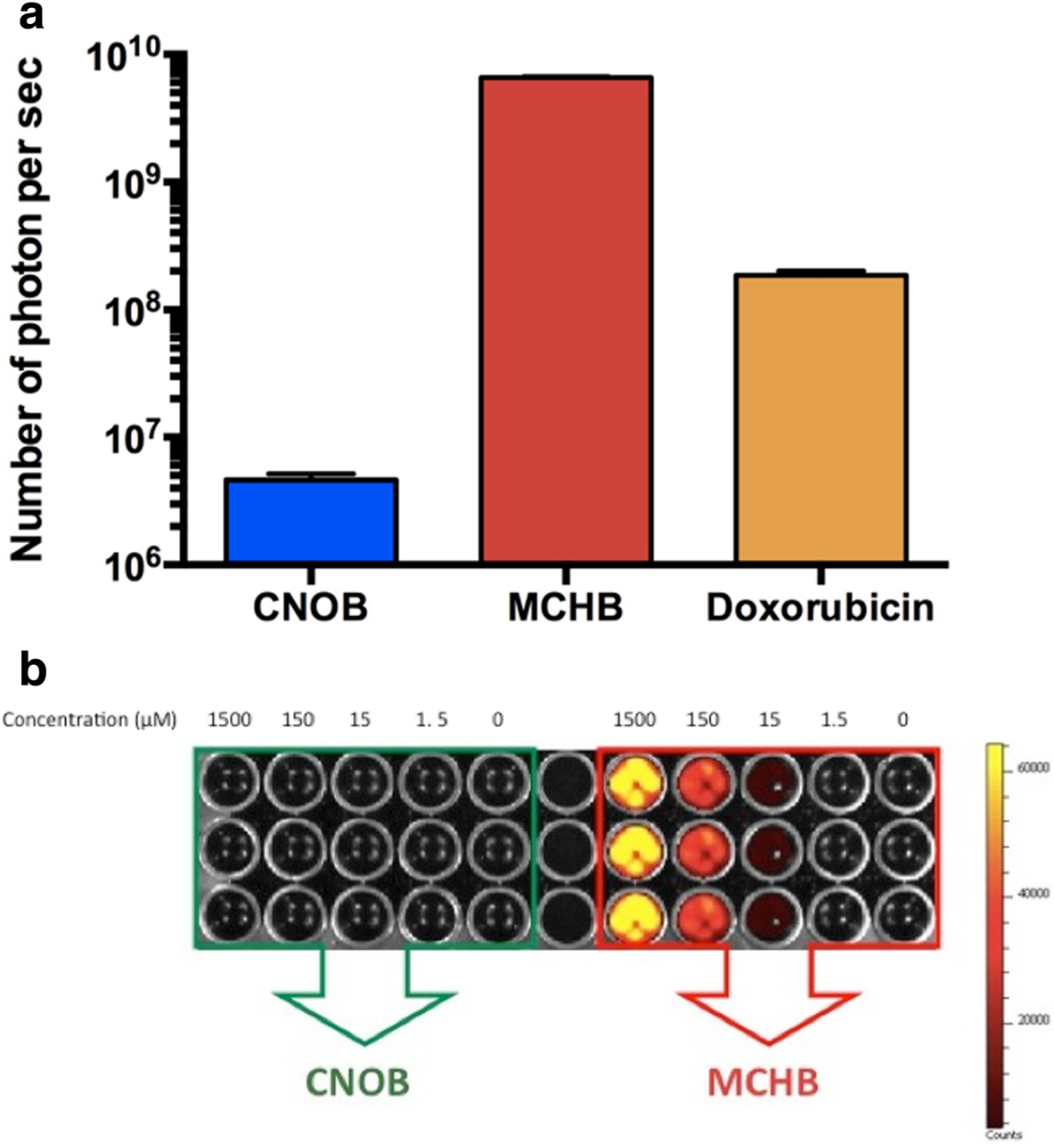
Fluorescence intensity of CNOB, MCHB and Doxorubicin. **a** Photon yields of CNOB (excitation/emission, 500/560 nm), MCHB (570/620 nm) and Doxorubicin (500/560 nm); all drugs were dissolved in DMSO at 15 μM concentration. **b** At MCHB fluorescence regimen (570/620 nm), CNOB fluorescence is negligible and thus does not interfere with fluorescence-based assessment of MCHB levels. The right bar indicates fluorescence intensity

In addressing this, we first examined if CNOB, having a nitro-substituted benzene ring, is itself fluorescent and thus might interfere with MCHB fluorescence. (CNOB and other compounds studied here were dissolved in DMSO at 15 μM concentration.) CNOB is indeed fluorescent, but its fluorescence properties are distinct from MCHB in both excitation and emission wavelengths: 570/620 nm for MCHB vs. 500/560 nm for CNOB; furthermore, CNOB fluorescence is much weaker than of MCHB, and at the peak emission wavelength of MCHB, CNOB fluorescence is negligible (Fig. 2). Doxorubicin, a widely used and well characterized anticancer drug, is also fluorescent and this property has facilitated its PK and other studies [16–20]. We found that MCHB generates over 15-fold greater number of photons than Doxorubicin (Fig. 2), making its fluorescence an important asset in characterizing and enhancing its therapeutic potential.

**Fig. 3:**
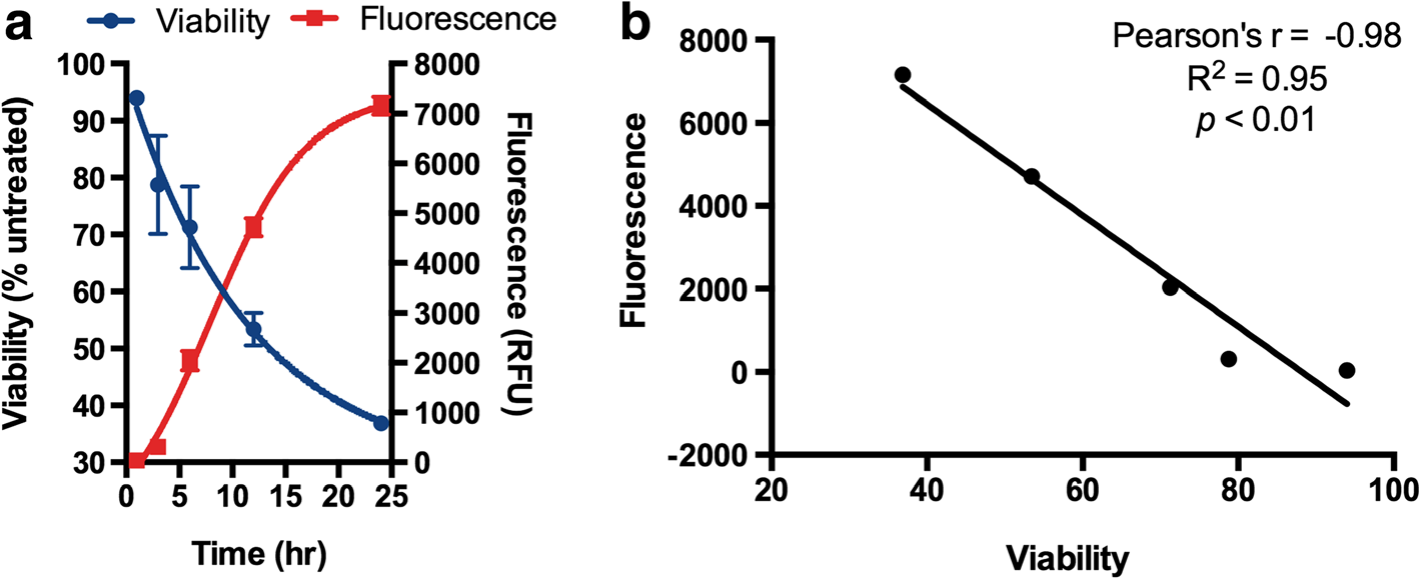
Correlation between MCHB fluorescence and cell killing in vitro. **a** MCHB fluorescence [relative units (RFU)] is shown in relation to loss of cell viability with time after CNOB (15 μM) addition to 4T1 cells constitutively generating HChrR6. Nonlinear regression was used for curve fitting. Error bars represent standard deviation (SD; *n* = 4). **b** Using GraphPad Prism software, correlation between viability and fluorescence (RFU) at each time point was calculated and is presented as Pearson’s *r* value and probability of correlation (*p* value)

That MCHB fluorescence might represent a quantitative measure of this drug was first suggested by in vitro studies. CNOB was added to transfected 4T1 cells endogenously expressing HChrR6, and cell killing and MCHB fluorescence were measured. A direct correclation was found between the fluorescence intensity and cell killing kinetics (Fig. 3).

**Fig. 4:**
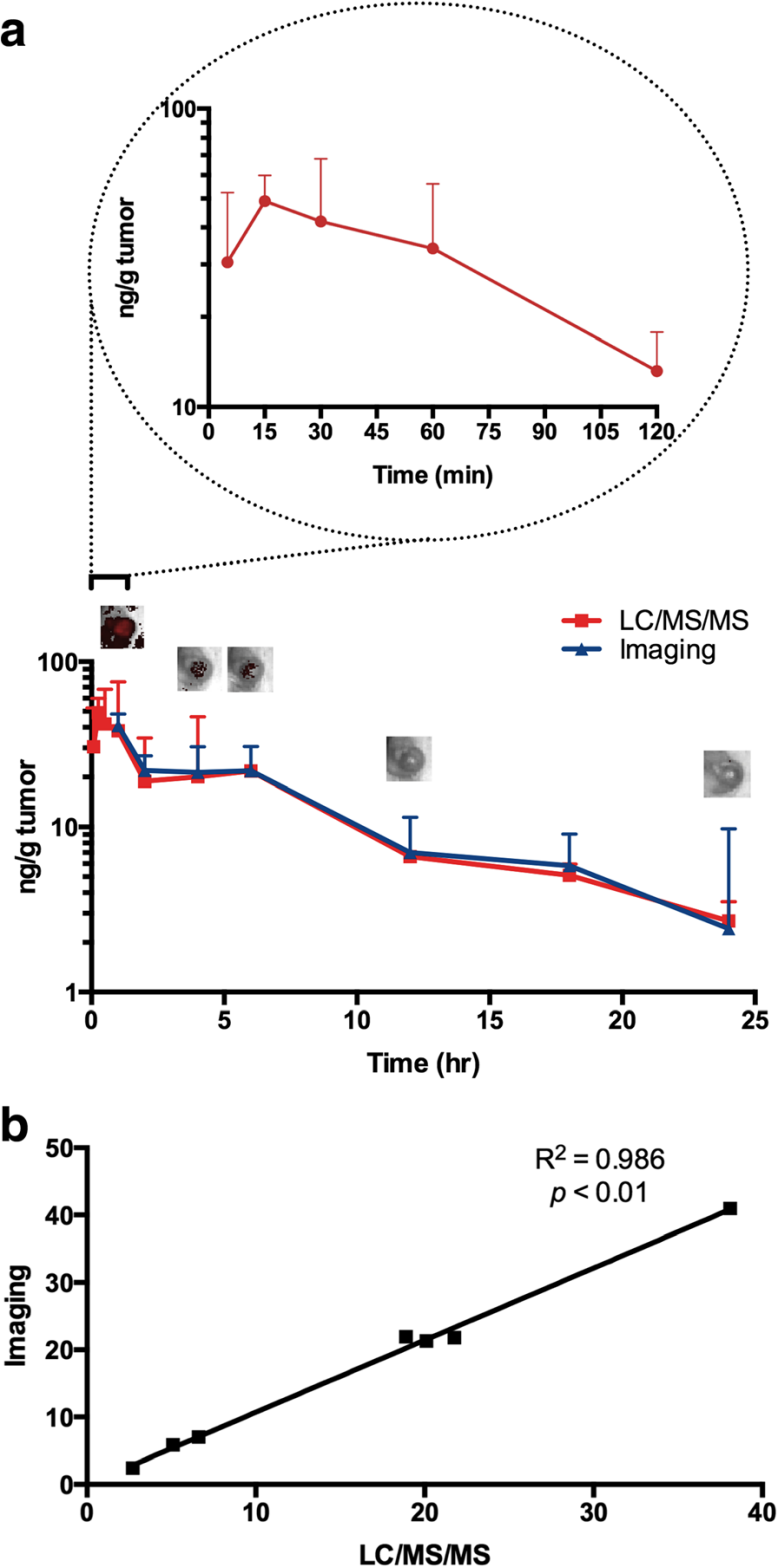
Quantification of MCHB in 4T1 tumors by imaging and LC/MS/MS. **a** The upper left encircled figure focuses on changes within the first two hours. Representative tumor images (above the curves) at the indicated time points illustrate the change in MCHB fluorescence, which was used to calculate MCHB concentration using a standard curve. (Fluorescence in the 12 and 24 h images is not evident to the naked eye but is recorded by the camera; see text for further details. At 24 h, the concentration of MCHB is extremely low, which accounts for the high variance at this time point; at other time points, the variance is markedly lower). **b** Correlation analysis of the two measurements: Pearson’s *r* = 0.99, *p* < 0.0001

To test if this might be the case also *in vivo*, orthotopic tumors were implanted using transfected 4T1 cells (endogenously expressing HChrR6) on mammary fat pad number 9 of mice, and CNOB (3.3 mg/kg) was injected via the tail vein. MCHB fluorescence was imaged in live mice (*n* = 4) and converted to its concentration using the standard curve mentioned in Materials and Methods (Fig. 4a). MCHB fluorescence could be imaged in the tumors as early as 5 min after CNOB injection, reaching a peak concentration of ca. 50 ng/g tumor at 15 min; at 24 h, the concentration had gone down to some 2 ng/g tumor, which could still be successfully imaged; the baseline count values were consistent overtime. (Note that fluorescence in the 12 and 24 h images is not evident to the naked eye but is recorded by the camera, being 33,700 ± 11,996 and 8,520 ± 1,698 counts/s, respectively). In another set of animals, MCHB in the tumors was measured by postmortem LC/MS/MS analysis (*n* = 4) at each of the above time points (Fig. 4a). Although this comparison involved separate sets of tumors in different mice, it is evident that the two methods nevertheless gave similar results: correlation analysis gave Pearson’s *r* value of 0.986 (*p* < 0.01; Fig. 4b).

Analogous experiments indicated that in plasma as well, imaging and LC/MS/MS analyses give similar results for MCHB concentration (Additional file 1: Figure S2). The results support the conclusion that measuring MCHB concentrations by fluorescence and LC/MS/MS give very similar values.

### Tumor PK measurements

Based on FDA recommendations [21], the PK parameters were analyzed at the single dose used above (3.3 mg/kg). Area under the tumor concentration curve (AUC) over the 24 h time course (AUC_0-24_) was calculated using the trapezoidal rule from the results of each MCHB measurement method from the data of Fig. 4a. Similar results were obtained: 325 ± 121 (h•ng/g) for LC/MS/MS and 336 ± 183 (h•ng/g) for imaging (Table 1).

**Table 1:** MCHB AUC in untransformed and *HchrR6*-transformed 4T1 tumors. AUC values in tumors were calculated over 24 h of CNOB treatment as determined by fluorescence imaging and LC/MS/MS. Statistical analysis of AUC was carried out by log transformed raw AUC data followed by two-tail paired *t*-test between each two groups of samples

AUC (h*ng/g tissue)	Imaging	LC/MS/MS
4T1	54 ± 34	50 ± 28
4T1/*HchrR6*	336 ± 183**	325 ± 121**

** *p* < 0.01 as compared between 4T1 and 4T1/*HchrR6* tumors using the same quantitative approach; no significant difference was observed between groups with the same type of tumors

As noted above, several nitroreductases, including the mammalian NQO1, are upregulated in cancer cells [2, 3] and our finding that CNOB alone has little anticancer effectiveness in mice [11] suggests that these enzyme levels must be ineffectual in activating this prodrug for treatment purposes. To gain an idea of these levels, we measured MCHB generation in ‘naïve’ tumors (i.e., tumors not generating HChrR6), using both fluorescence- and LC/MS/MS-based quantification methods. These tumors did generate MCHB, but to an AUC_0-24_ which was some six-fold less than the transfected tumors (Table 1). As no curative effect of CNOB was seen in mice implanted with the naïve 4T1 tumors (the survival was equal to PBS injection) -- the AUC_0-24_ exposure levels of the naïve and tranfected tumors bracket the non-curative and significantly curative MCHB levels: the survival of mice with transfected tumors at this dose increased by 45 days [11]. The results of Table 1 again show that MCHB quantification by imaging and LC/MS/MS give similar values.

### Plasma PK measurements

**Fig. 5:**
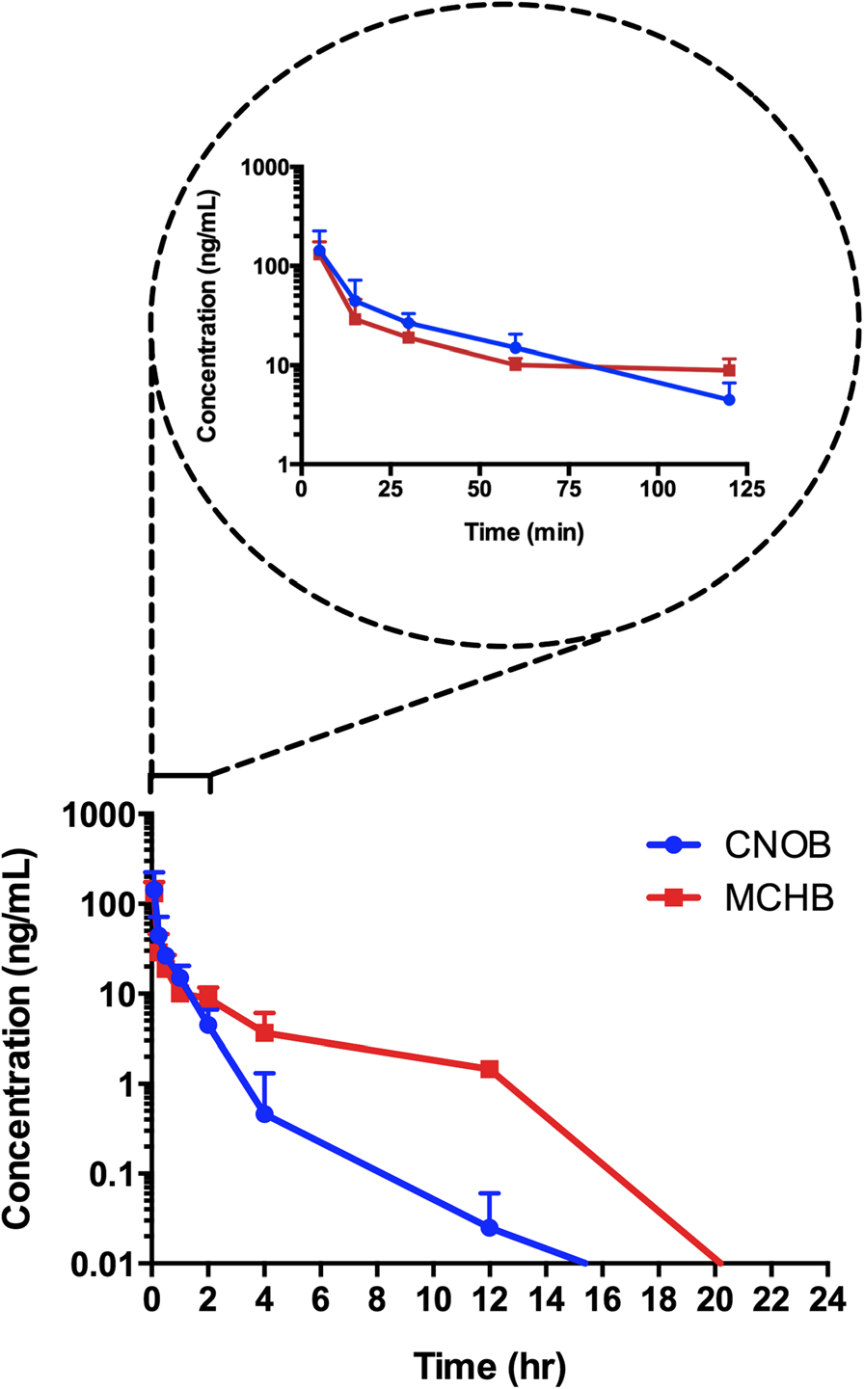
Plasma levels of MCHB and CNOB in mice bearing 4T1 orthotopic tumors expressing HChrR6. The upper left encircled figure focuses on changes within the first two hours. Following tail vein CNOB injection plasma was harvested at various time points. Plasma levels of MCHB and CNOB were quantified by LC/MS/MS. The lower figure provides values up to the time points where the concentration of the compounds could be reliably measured. Note that the CNOB and MCHB concentrations are shown as touching the abscissa; this is because the measured quantities at 24-h time point were below the detection limit of the instrument (We did obtain the LC/MS/MS quantification data for the 24-h time point. But since it was below the instrument’s quantification limit). Plasma was collected at indicated times after CNOB injection (3.3 mg/kg). Data represent average of samples at each time point (*n* = 4). See text for further details

Plasma PK parameters were estimated ex vivo for both MCHB and CNOB by LC/MS/MS analysis (Fig. 5). The data were analyzed using non-compartmental model (Table 2). Peak plasma concentrations (C_max_), and exposure (AUC_0-24_), were similar for CNOB and MCHB. Volume of distribution was estimated to be high for both CNOB and MCHB at 81.5 and 117 L/kg, respectively, suggesting extensive extravascular distribution, which agrees with previous findings [11]. As regards the terminal phase parameters, the clearance (CL) values were similar, and although the half-life tended to be different (t_1/2_: CNOB 4.6 h, MCHB 8.3 h), both were in the shorter range. It should be noted that the MCHB parameter estimates are influenced by the kinetics of CNOB conversion to MCHB. Although the densities of the tumors and plasma differ, it is apparent in comparing AUC values that there was a considerably greater amount of tumor exposure to MCHB, which is consistent with the curative effect at this CNOB dose mentioned above.

**Table 2:** Plasma pharmacokinetic parameters of MCHB and CNOB in mice carrying 4T1/*HchrR6* tumors. Plasma samples were collected at various times (5 min, 15 min, 30 min, 1 h, 2 h, 4 h, 6 h, 12 h, and 24 h) after iv dose of CNOB in nude mice carrying 4T1*/HchrR6* tumors. MCHB and CNOB levels overtime were determined by LC/MS/MS

CNOB Dose	Analyte	C_max_ ± SE	AUC_0-24_ ± SE	V_ss_	CL	t_1/2_
mg/kg		ng/mL	h*ng/mL	L/kg	L/h/kg	h
3.3	CNOB	141 ± 28.6	166 ± 23.5	81.5	17.7	4.6
	MCHB	145 ± 30.4	148 ± 16.0	117	21.1	8.3

*C*_*max*_ maximum observed concentration ± standard error (SE), *AUC*_*0-24*_ area under the concentration curve from 0 to 24 h ± SE, *V*_*ss*_ volume of distribution, *CL* clearance; *t*_*1/2*_ terminal elimination half-life

### Prediction of a more effective dosage regime

In further preclinical studies, we are currently attempting to use extracellular vesicles (also referred to as exosomes [22]) to specifically deliver CNOB activating capacity to HER2 +ve breast cancer and to develop a more curative therapeutic regimen. These studies would be facilitated by guidance on alternate doses/administration schedules of CNOB that may enhance its therapeutic efficacy.

This analysis required assumption of PK linearity. We reasoned that this assumption was justified given the following facts. The *in vivo* activation of CNOB into MCHB took place within a very short time (five minutes; Fig. 4); the same was the case with its clearance, since we could detect MCHB in urine and feces of the mice within 15 min of CNOB injection (data not shown), and little MCHB could be detected in the tumor by 24 h (Fig. 4). The PK profile of both CNOB and MCHB in the plasma was also very rapid as neither compound remained in the plasma by 24 h. Therefore, at our multiple dosing schedule of 48 h, the clearance from the previous dose was complete.

**Fig. 6:**
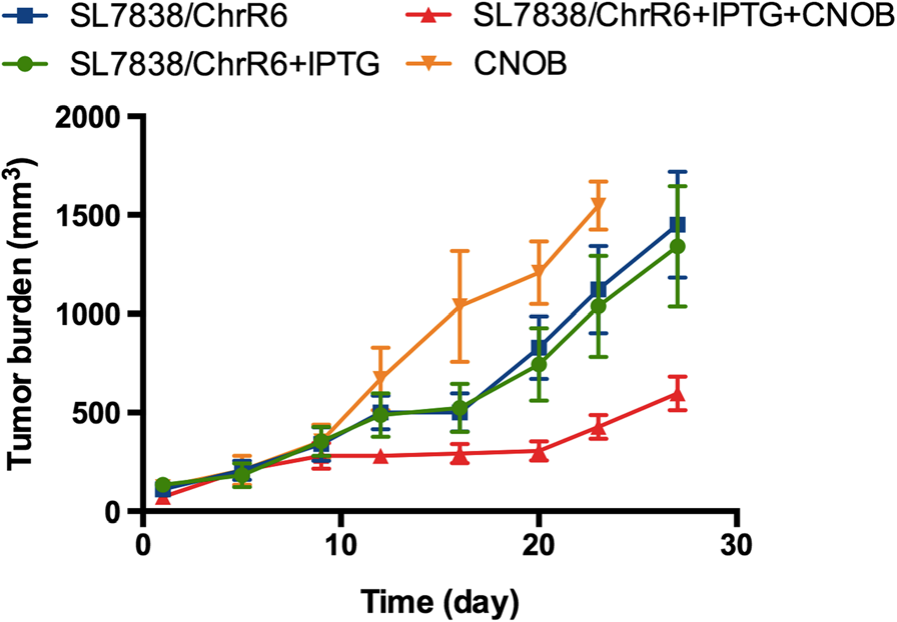
Tumor burden in implanted 4T1 murine mammary tumors following CNOB administration via tail vein. ChrR6 was delivered iv using SL7838 bacteria carrying the gene encoding this enzyme, controlled by the *lac* promoter; IPTG was used when activation of the gene was required. Tumor burden was measured on the indicated days following CNOB injection (10 mg/kg in three 3.3 mg/kg daily doses). Data represent mean value ± standard deviation (SD) (*n* = 5). See Figure for the symbols

**Fig. 7:**
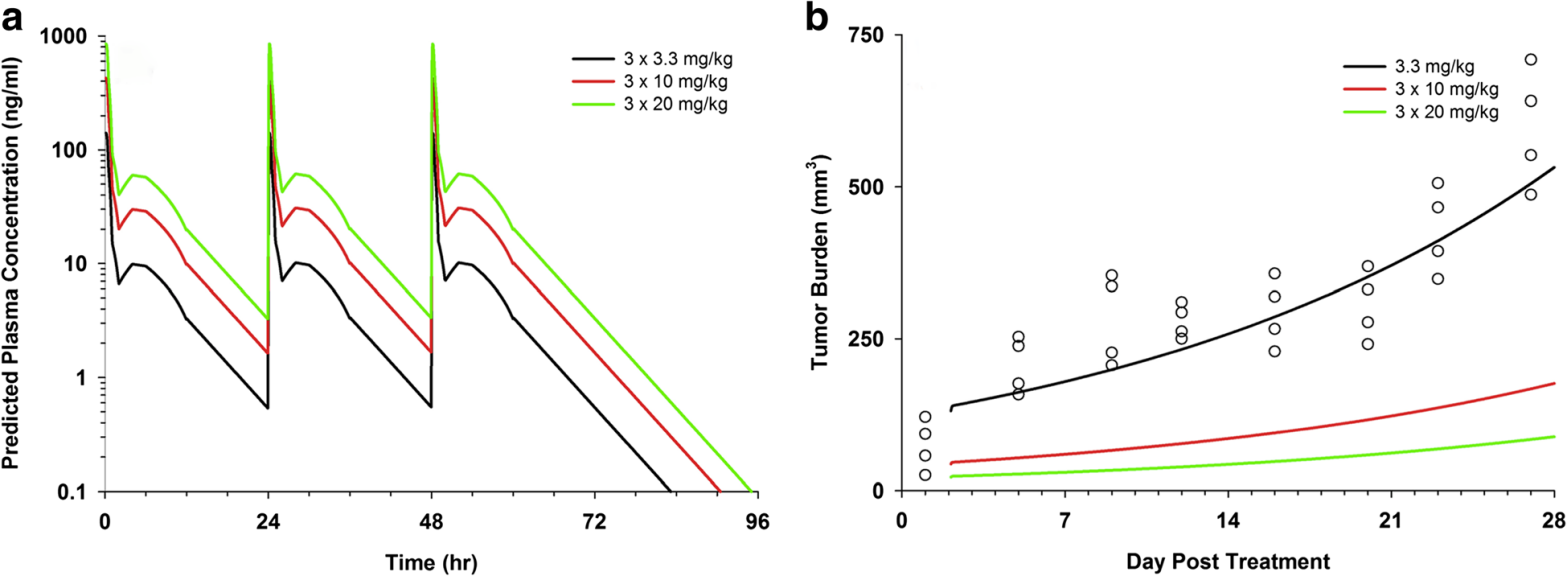
Simulation of CNOB dose administrations and predicted tumor growth inhibition. Based on the PK parameters estimates from noncompartmental analysis of single (3.3 mg/kg) dose of CNOB, and tumor growth data (Fig. 6), a combined PK/PD model was constructed which included a 2 compartment iv model linked with an inhibitory E_max_ model. Simulation predicted plasma CNOB concentrations (**a**) and corresponding tumor growth curves (**b**) after the indicated daily doses of CNOB. Circles represent original tumor burden data from Fig. 6

Using the PK parameters estimated above for the single (3.3 mg/kg) dose as starting point, tumor growth kinetics at the dosage regime used in our previous study (10 mg/kg total, administered in three daily doses of 3.3 mg/kg [11]; Fig. 6), and assuming PK linearity as mentioned above, we constructed a combined PK/PD model. Non-compartment PK parameters were used as initial estimates in the combined PK/PD model, which includes a two compartment iv (PK) model linked with an inhibitory E_max_ (PD) model. Figure 7 shows the predicted plasma CNOB concentrations (**a**) and corresponding tumor growth curves (**b**) after simulation of 3.3, 10, and 20 mg/kg of CNOB given daily for 3 days. Based on the tumor growth curves, these simulations suggest that increasing doses of CNOB may have notable effects on tumor progression, and large increases in CNOB dosage may not be required for successful therapy.

## Discussion

The fluorescence of CNOB is negligible at the emission wavelength of MCHB, meaning that the fluorescence thus measured is that of MCHB alone. Further, measurements of MCHB concentration by its fluorescence and by LC/MS/MS gave similar results. This was shown in vitro by cell killing kinetics, and in vivo for both transfected and untransfected implanted 4T1 tumors (with and without endogenous HChrR6 expression) down to MCHB levels of 2 ng/g tumor, as well as for the plasma. We conclude that the non-invasive MCHB fluorescence imaging is a reliable indicator of its concentration.

As mentioned in the Background, certain conditions must be met for successful development of an F-prodrug regimen, and the fluorescence characteristics identified here provide a powerful tool for attaining these conditions for the CNOB-GDEPT. Two examples will suffice to highlight the importance of this tool in this context. First, to attain specific targeting that confines the CNOB-activating capability to the tumor would require testing a variety of methods involving the use, for instance, of different delivery vehicles, and targeting ligands for a given cancer. As Additional file 1: Figures S1 and S2 illustrate, noninvasive imaging of MCHB fluorescence in living mice can provide a rapid screen of the relative success of methods differing in these, as well as other aspects, that may need to be tested for specific targeting of the tumor. Of course, final confirmation will necessitate the use of *ex-situ* and more robust methods, such as LC/MS/MS, immunohistochemical, Western and others, but a quick initial ‘observational’ screen will greatly narrow the outcomes that would require the use of these involved and labor intensive techniques.

The second example that illustrates the advantage of MCHB fluorescence concerns the fact that gene expression has proved a limiting factor in the success of F-prodrug therapy (Background). Several different approaches would need to be tested to address this problem. Although DNA has been primarily used in gene delivery, there may be compelling advantages in using mRNA instead. For DNA-mediated gene delivery, transport into the nucleus is required for expression, and It is well established that DNA transport to the nucleus is highly inefficient [23, 24]; in contrast, mRNA expression can occur directly in the cytosol. Studies have indeed shown the superiority of mRNA over DNA in gene transfer in both proliferating and non-proliferating cells [25, 26]. Direct protein transfer may also need to be considered along with measures to enhance the stability and duration of expression. As is seen in Fig. 4, the relative effectiveness of these approaches in improving the level and duration of expression of the gene (or mRNA) and its peak levels can also be quickly gauged with this prodrug regimen by imaging, minimizing the need for the *ex situ* involved techniques. Visualization approaches can also be applied to rapidly assess the extent of transfection of cells in tumors to generate the bystander effect required for effective therapy.

The PK parameters measured here enabled us to predict ways of administering CNOB to make the therapy more effective. This information will aid in the clinical transfer of this regimen. Indeed, we have already succeeded in specific delivery of ChrR6 mRNA to the HER2 +ve BT474 cells conferring on them the capacity to convert CNOB into MCHB (ms in preparation). Further studies for transfer to the clinic of the exosome-based regimen will utilize more sophisticated PK/PD models, for example that developed by Simeoni et al (http://www.ncbi.nlm.nih.gov/pubmed/14871843) to link plasma concentration over time data to tumor growth.

## Conclusion

The fluorescence intensity of the cytotoxic product of CNOB-GDEPT, MCHB, quantitatively reflects its exposure level in the tumor and plasma. This feature provides a powerful tool to rapidly screen a variety of approaches to make this regimen a successful anticancer therapy; it permits noninvasive imaging of MCHB generation in live mice, thereby greatly narrowing the outcomes that would need to be followed up by the use of more rigorous but also more labor intensive approaches. The prediction of a more optimal dose regimen of CNOB reported here will also facilitate attaining this end.

## Abbreviations

AUC, area under the curve; AUC_0-24_, AUC between 0 and 24 h; BE, bystander effect; CB1954, 5-aziridinyl-2,4-dinitrobenzamide; ChrR, chromate reductase; ChrR6, improved form of ChrR; CL, clearance; C_max_, maximum observed concentration; CNOB, 6-chloro-9-nitro-5-oxo-5H-benzo-(a)-phenoxazine; DMEM, Dulbecco’s modified eagle medium; E_max_, maximum effect attributable to the drug; F-Luc, firefly luciferase; F-prodrugs, prodrugs requiring a foreign enzyme for activation; GC, Gencicloir; GDEPT, gene-delivered enzyme prodrug therapy; HER2-positive breast cancer, breast cancer overexpressing human epidermal growth factor recptor 2; ip, intraperitoneal; IPTG, isopropyl β-D-1-thiogalactopyranoside; iv, intravenous; LC/MS/MS, liquid chromatography/tripe quadruple mass spectroscopy; Luc, luciferase; MCHB, 9-amino-6-chloro-5H-benzo[a]phenoxazine-5-one; MEM, minimum essential medium; MMC, Mitomycin C; N-prodrugs, prodrugs activated by native tumor enzymes; NQO1, another name for mammalian NAD(P)H quinone dehydrogenase 1; NTR, bacterial nitroreductase; PK/PD, pharmacokinetics/pharmacodynamics; RFU, relative fluorescent units; SD, standard deviation; SE, standard error; t_1/2_, half life; TK1, herpes simplex virus 1 thymidine kinase; USFDA, United States Food and Drug Administration; V_ss_, volume of distribution at steady state

## Additional file

Additional file 1:
**Figure S1.** Visualization of MCHB generation in living mice by fluorescence imaging. Following CNOB administration, MCHB fluorescence was visualized by imaging in implanted murine mammary tumors expressing firefly luciferase (F-Luc; for bioluminescent imaging); ChrR6 was delivered intratumorally using bacteria carrying the gene encoding this enzyme and expressing Lux to visualize them. (The Luc signal includes Lux, but because the former was >50-fold greater, the latter is negligible.) IVIS (bioluminescence) and Maestro (fluorescence) systems were used in imaging. Reproduced from our previous work [11] for ease of reference. **Figure S2.** Correlation between plasma MCHB levels determined by fluorescence and LC/MS/MS measurements. MCHB was measured in the plasma of mice bearing implanted 4T1 tumors expressing HChrR6 by fluorescence imaging or LC/MS/MS at selected time intervals following tail vein injection with CNOB (3.3 mg/kg). Quantification was done as described in Materials and Methods. Statistical analysis was performed using GraphPad Prism. (DOCX 536 kb)
