# Supplementary material for: Utilizing native fluorescence imaging, modeling and simulation to examine pharmacokinetics and therapeutic regimen of a novel anticancer prodrug
Source: BMC Cancer. 2016 Jul 25;16:524. doi: 10.1186/s12885-016-2508-6 (PMC4960810; doi:10.1186/s12885-016-2508-6)
Supplement: Additional file 1: — Figure S1. Visualization of MCHB generation in living mice by fluorescence imaging. Following CNOB administration, MCHB fluorescence was visualized by imaging in implanted murine mammary tumors expressing firefly luciferase (F-Luc; for bioluminescent imaging); ChrR6 was delivered intratumorally using bacteria carrying the gene encoding this enzyme and expressing Lux to visualize them. (The Luc signal includes Lux, but because the former was >50-fold greater, the latter is negligible.) IVIS (bioluminescence) and Maestro (fluorescence) systems were used in imaging. Reproduced from our previous work [11] for ease of reference. Figure S2. Correlation between plasma MCHB levels determined by fluorescence and LC/MS/MS measurements. MCHB was measured in the plasma of mice bearing implanted 4T1 tumors expressing HChrR6 by fluorescence imaging or LC/MS/MS at selected time intervals following tail vein injection with CNOB (3.3 mg/kg). Quantification was done as described in Materials and Methods. Statistical analysis was performed using GraphPad Prism. (DOCX 536 kb) [file 12885_2016_2508_MOESM1_ESM.docx]

**Additional file 1**


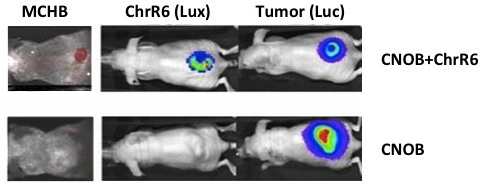


**Figure S1. Visualization of MCHB generation in living mice by fluorescence imaging.** Following CNOB administration, MCHB fluorescence was visualized by imaging in implanted 4T1 murine mammary tumors expressing firefly luciferase (F-Luc; for bioluminescent imaging); ChrR6 was delivered intratumorally using bacteria carrying the gene encoding this enzyme and expressing Lux to visualize them. (The Luc signal includes Lux, but because the former was >50-fold greater, the latter is negligible.) IVIS (bioluminescence) and Maestro (fluorescence) system were used in imaging. Reproduced from our previous work [11] for ease of reference.


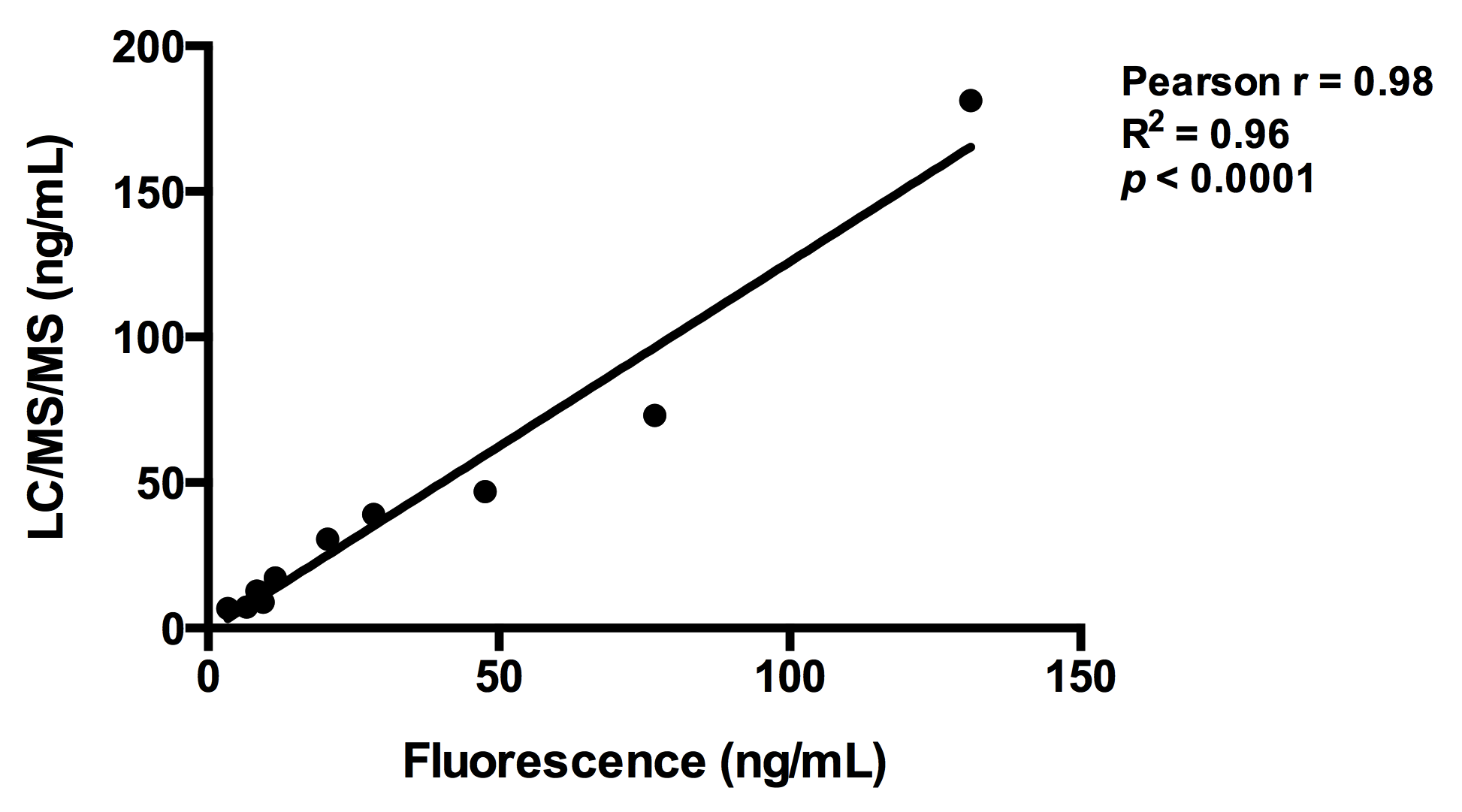


**Figure S2. Correlation between plasma MCHB levels determined by fluorescence and LC/MS/MS measurements.** MCHB was measured in the plasma of mice bearing implanted 4T1 tumors expressing HChrR6 by fluorescence imaging or LC/MS/MS at selected time intervals following tail vein injection with CNOB (3.3 mg/kg). Quantification was done as described in Materials and Methods. Statistical analysis was by GraphPad Prism.
